# The salience of self, not social pain, is encoded by dorsal anterior cingulate and insula

**DOI:** 10.1038/s41598-018-24658-8

**Published:** 2018-04-18

**Authors:** Irene Perini, Per A. Gustafsson, J. Paul Hamilton, Robin Kämpe, Maria Zetterqvist, Markus Heilig

**Affiliations:** Department of Clinical and Experimental Medicine, Center for Social and Affective Neuroscience, Linköping University, 581 83 Linköping, Sweden

## Abstract

The human neural correlates of social rejection have attracted significant research interest, but remain subject to vigorous debate. Specifically, it has been proposed that a matrix of brain regions overlapping with the classical pain matrix, and including the dorsal anterior cingulate cortex (dACC) and the anterior insular cortex (AI) is critical for processing of social rejection. The present study expands on this conceptualization, by showing that these areas are involved in processing of self-relevant social evaluation, irrespective of valence. Forty healthy adolescents (N = 20 females) were tested in a magnetic resonance imaging (MRI) scanner. We used a novel paradigm that balanced participants’ experience of rejection and acceptance. In addition, the paradigm also controlled for whether the social judgment was towards the participants or towards other fictitious players. By creating a “self” and “other” distinction, we show that right AI and dACC are involved in processing the salience of being judged by others, irrespective of the quality of this judgment. This finding supports the idea that these regions are not specific to social rejection or even to pain or metaphorically painful experiences, but activate to self-relevant, highly salient information.

## Introduction

Social interactions are critical for health and well-being in humans and other group-living primates. Adolescence is a particularly sensitive period in this regard, with peer-relations becoming increasingly important during this developmental stage^1,2^. The desire for peer approval plays an important role in identity development and self-esteem during adolescence^1,3^. The transition from childhood to adolescence is also accompanied by unique cerebral changes. Regions involved in executive control processes such as response inhibition and working memory, mature late in adolescence. Neurodevelopmental changes are not limited to prefrontal regions but are widespread in the brain. Recent evidence highlights the relationship between networks, suggesting that during adolescence cross-network interaction becomes more specialized^4^. These networks, which are well characterized in adults, support executive control, internalization, and sustained attention. Without fully-developed strategies for regulating affective experiences or evaluating risk, adolescents can be considered especially vulnerable to social adversities, such as negative social evaluation and peer rejection^5^.

The ability to evaluate information that signals social acceptance or rejection is critical for successful socialization. Social rejection is associated with detrimental emotional^6,7^ and cognitive^8^ effects. Understanding its impact on the brain is therefore an important research goal for social neuroscience, and has broader implications for mental health research^9^.

Simulating social interactions in a functional neuroimaging environment requires a trade-off between real-world validity and experimental control, posing a challenge to research aimed at identifying neural mechanisms of social rejection and its consequences. One of the most widely used approaches to address this challenge is the Cyberball task. In this paradigm, participants engage in a ball-tossing game with two simulated online players. During the game, participants are initially included in the ball-tossing, but then become increasingly excluded^10^. This task has shown strong construct validity, as it consistently induces negative emotional states following rejection^11–13^. The Cyberball task has been widely used in functional magnetic resonance imaging (fMRI) investigations of social rejection^14–17^.

A pioneering study that used the Cyberball task reported activation of the dorsal anterior cingulate cortex (dACC) and anterior insula (AI) during epochs of rejection. Moreover, this dACC activation was found to be correlated with self-reported levels of distress in response to rejection^17^. As AI and dACC activity is classically observed in response to physical pain, it was therefore suggested that physical and psychological pain share brain structures^18^. This conceptualization has a strong intuitive appeal, in part because many languages use metaphors of physical pain to express the subjective experience of interpersonal loss and rejection^19^. Physical and social pain also engage some of the same endocrine mediators; for instance, endogenous opioids and oxytocin have analgesic effects^20–22^ but also mediate attachment behaviors^23–25^.

Subsequent research has, however, led to a vigorous debate regarding the relation between the neural substrates of physical and social pain. While it is clear that social processes can result in activation of structures that overlap with “the physical pain matrix”—i.e. the AI and dACC—more recent data have called into question both the extent of this overlap^14–16^, and also whether it is specific for the experience of rejection^26–30^.

AI and dACC are involved in multiple, complex cognitive functions. In particular, since the pioneering studies of Eiseberger *et al*.^17^, it has become clear that AI and dACC are key nodes of the salience network^31,32^, a set of interconnected brain structures involved in switching between internal and externally directed psychological processes^33,34^. Balancing the salience of experimental conditions has therefore become crucial for understanding the specificity of brain responses to social interactions. A recent study capitalized on a design in which negative and positive social feedback was made equally salient. Prior to the scan, the participants made a short video in which they talked about themselves, and this was then evaluated by a panel of judges while the subject was in the scanner. Using a conjunction analysis, the authors found that the AI and dACC were activated by both positive and negative feedback^35^. This finding illustrates the importance of balanced conditions in fMRI paradigms aimed at identifying neural substrates of social evaluation. However, the specific role of these structures in social interactions remains unknown and discerning this role requires more explicit experimental questions.

**Figure 1:**
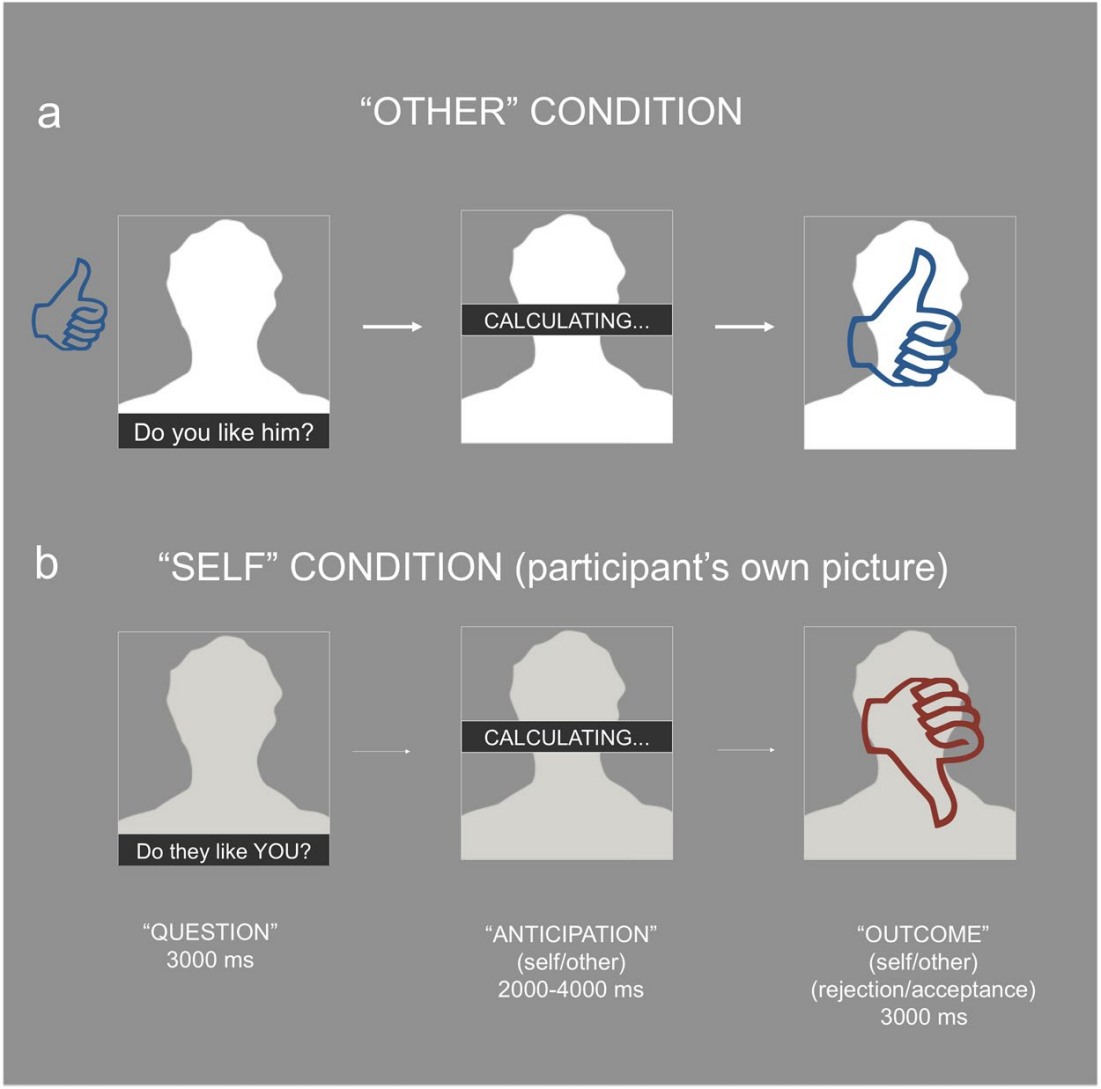
Design description. (**a**) Chronological sequence of the “other” trials. (**b**) Chronological sequence of the “self” trials. Neutral frontal face images were used in the experimental procedure for both self and other conditions. The ITI lasted 2000, 3000 or 4000 milliseconds. ©Yvwv/Wikimedia Commons/https://creativecommons.org/licenses/by-sa/3.0/ ©Debivort/Wikimedia Commons/https://creativecommons.org/licenses/by-sa/3.0/.

Here, we addressed the hypothesis that the salience of social evaluation may be critically determined by a “self” versus “other” dimension; and that AI and dACC responses to social feedback may be involved in encoding this dimension. To test this hypothesis, we applied a novel paradigm that allowed us to examine the interaction of social evaluation, balanced across the spectrum of rejection and acceptance, with whether the social judgment was directed towards the participant or other players. Forty healthy teenagers engaged in a simulated online game during fMRI scanning. Participants viewed briefly-presented pictures of other players’ faces, one at the time, and were asked to indicate whether they liked the other player or not. Following this, a cue signaling the collective outcome was superimposed on the picture of the other player. Similarly, participants viewed pictures of their own faces and were judged by other players during a given trial (see Fig. 1). Using this approach, we were able to isolate the brain correlates of positive or negative social feedback from those of self-referential processing. Finally, because of an increasing realization of a potential for sex differences in brain function^36^, we ensured that males and females were equally represented in our study, allowing us to carry out a secondary analysis that explored potential sex differences in brain responses to social evaluation.

## Results

### Behavioral findings

On average our participants correctly estimated that they were disliked in about half of the trials, showing no attentional bias towards negative or positive feedback [51.4 ± 15.5, mean% ± SD; no sex differences; *t*(36) = −0.89, *p* = 0.38]. On average, they also accorded an equal proportion of likes and dislikes towards other players [44.3 ± 17.9, mean% ± SD, range 0–78%; no sex differences were observed for this behavioral outcome; *t*(38) = 0.27, *p* = 0.78)]. When receiving “likes” participants indicated that they felt good [mean ± SD = 5.63 ± 2.85 on a 10-point “feel good scale,” where neutral = 0; *t*(37) = 12.17, *p* < 0.001]. When receiving a “dislike” the participant rated that they were feeling bad [mean ± SD = 3.56 ± 2.81 on the 10-point “feel bad scale,” where neutral = 0; *t*(37) = 7.82, *p* < 0.001]. No sex differences were observed for these two measures; [*t*(36) = −0.91, *p* = 0.37; *t*(36) = −0.37, *p* = 0.71, respectively].

### Brain correlates of anticipating social evaluation

For the anticipation interval, we compared neural response for “self” versus “other” conditions. A whole brain within-subjects analysis showed an activation of the right AI and bilateral dACC for the “self” versus “other” conditions contrast. In addition, the supplementary motor area (SMA) and postcentral gyrus bilaterally (PCG) were also significantly activated in this contrast (*p < *0.01). No significant sex differences were found (see Table 1 for coordinates).

**Table 1:** Activations associated with the whole-brain analyses during the anticipation interval, expressed by peak scores in Talairach-space coordinates (x, y, z). Z scores survived significance threshold (alpha < 0.05, cluster corrected).

Analysis	Region	Talairach coordinates			voxels
		x	y	z	
Anticipation Interval					
Self > Other					
	SMA	2	−7	59	348
		−4	8	42	
	dACC	9	14	32	
	PCG	−37	−46	56	263
		38	−49	56	136
	SPL	−19	−70	44	63
		26	−70	44	63
	AI	35	17	11	87

AI = anterior insula; dACC = dorsal anterior cingulate cortex; PCG = precentral gyrus; SPL = superior parietal lobule SMA = supplementary motor area.

### Brain correlates of receiving social evaluation: self vs. other dimension

On brain response data taken during the outcome interval, we first performed a whole brain 2 × 2 factorial ANOVA with factors Perspective (two levels: self and other) and Outcome (two levels: rejection, acceptance). For the main effect of Perspective, the right mid-anterior insula and dACC, substantia nigra (SN), superior frontal gyrus (SFG) and posterior occipital cortex (POC) were significantly activated (*p* < 0.02).

**Figure 2:**
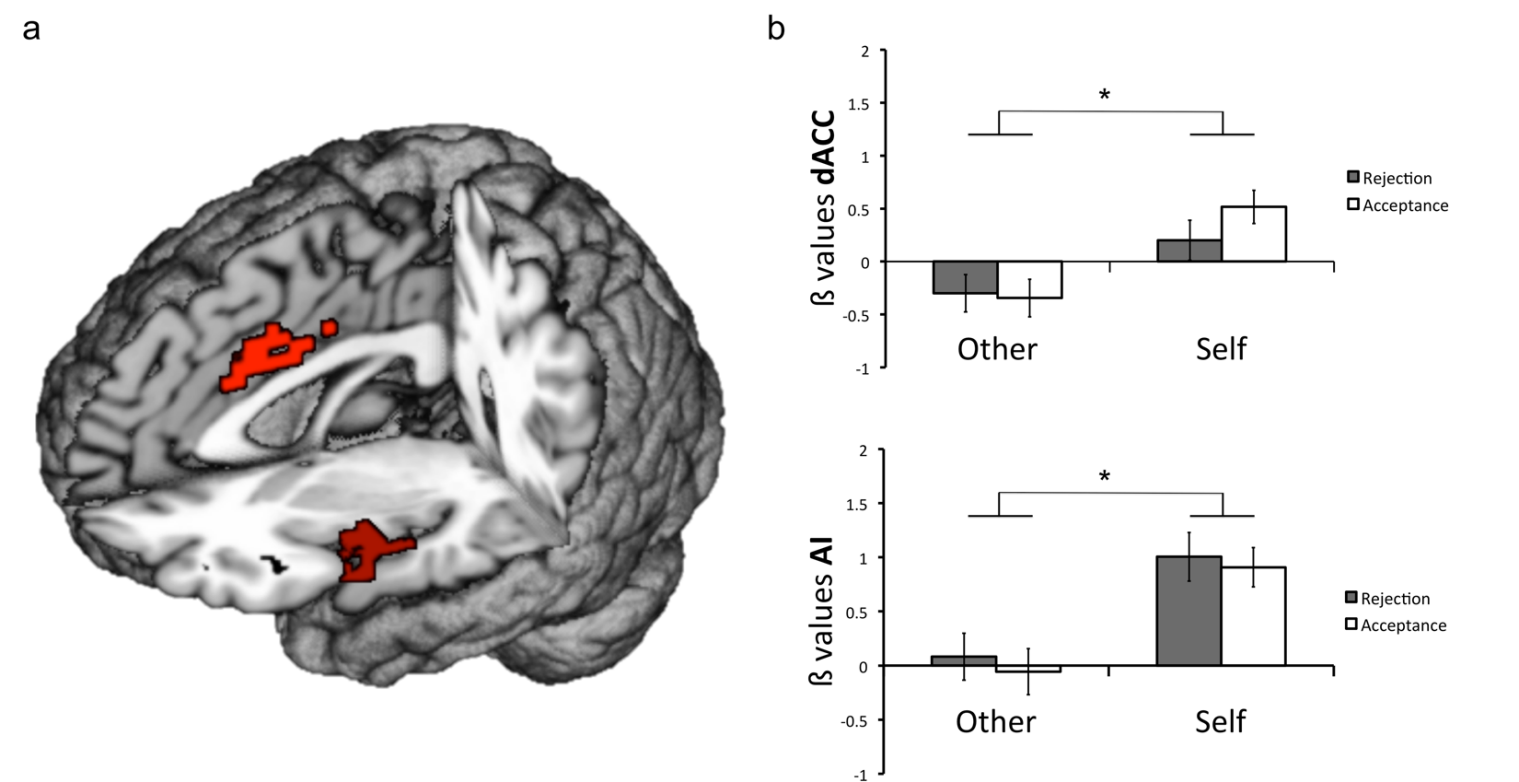
A 2 × 2 factorial ANOVA with factors perspective (2 levels: self and other) and outcome (2 levels: rejection and acceptance) was performed at the whole brain level. (**a**) Significant rAI and dACC activations for the factor perspective. (**b**) Bar graphs show significantly higher average β-values for “self” versus “other” conditions in dACC and AI. In rAI average β-values a perspective x gender interaction was observed. Error bars represent SEM.

We then extracted the ß values from the rAI and dACC clusters detected above, and performed a repeated measures 3-way ANOVA with Perspective and Outcome as within-subjects factors and Sex as a between-subjects factor. Within the right AI, this analysis showed a significant effect of Perspective with “self” condition values being higher than “other” condition values [*F*(1, 38) = 21.2, *p* < 0.001, *η*^2^_*p*_ = 0.36]. Although less robust, we also found a Perspective-by-Sex interaction in which females had significantly lower values than males for the other condition [*F*(1, 38) = 5.07, *p* = 0.03, *η*^2^_*p*_ = 0.12)]. Analysis of ß values extracted from the dACC showed results similar to those of the right AI. Specifically, there was a main effect of Perspective with “self” condition values higher than “other” condition values [*F*(1, 38) = 18.4, *p < *0.001, *η*^2^_*p*_ = 0.33] (Fig. 2). To test the possibility that this finding might be due to recognition of one’s own face we extracted and compared ß values from AI and dACC during the “question phase” (Fig. 1) at the beginning of each trial when participants processed their faces compared to other players faces. We observed no significant effect of Perspective (*p* > 0.05) during this phase of each trial.

We detected no Perspective-by-Outcome interaction in the dACC or AI; we did, however, observe such an interaction in right fusiform gyrus, left middle occipital gyrus, and precuneus. The rejection versus acceptance comparisons within both the “self” and ‘‘other’’ conditions did not exceed statistical threshold. Coordinates and spatial extents of all regional neural effects are listed in Table 2.

**Table 2:** Activations associated with the whole-brain analyses during the outcome interval, expressed in Talairach-space coordinates (x, y, z).

Analysis	Region	Talairach coordinates			voxels
		x	y	z	
Outcome Interval					
ME Perspective	POC	20	−76	−1	521
		−7	−70	8	
	SN	−4	−22	−10	
	SFG	31	36	47	102
	dACC*	−1	8	26	65
	mid/AI*	44	5	−1	64

Z scores survived significance threshold (alpha > 0.05, cluster corrected). POC = posterior occipital cortex; SN = substantia nigra; SFG = superior frontal gyrus; dACC = dorsal anterior cingulate cortex; mid/AI = anterior insula; ME = main effect. *Significantly higher activation for the self versus other conditions comparison.

## Discussion

Our study sheds new light on investigations of social rejection by balancing the salience of positive and negative social evaluations, and by including as an additional dimension whether social evaluation was directed toward the participant or others. To achieve this objective in a manner that is relevant for real-world conditions while maintaining experimental control, we designed a task that mimicked the social media environment in which Swedish adolescents and young adults spend extensive amounts of time^37^.

At a behavioral level, we found that being negatively judged by others led to reports of modest negative emotions, less pronounced than prior studies showing strong negative emotional effects^17,38,39^. We believe the lower magnitude of self-reported negative emotions is our study is due to the fact that our design is fundamentally different from previous studies that investigated social rejection. Our fast-paced, event-related design was aimed to trigger opposite emotions at short intervals at a trial-by-trial level. It is therefore not well suited to studying self-reported global emotional responses, since its goal is not to induce a general negative (or positive) feeling. Nevertheless, although less pronounced, our behavioral findings show that the task induced emotional responses consistent with the conditions.

The fMRI results show an informative set of determinants of dACC and right AI responses to social evaluation. These areas were activated during the “self” compared to the “other” condition, irrespective of the valence of social feedback. Interestingly, our results show that dACC and AI were significantly activated also during the anticipation phase, before the participant knew the nature of the social feedback to come. It is possible that the anticipation interval might reflect two different psychological states, since in the “self” condition the participant anticipates without the possibility to decide, whereas in the other condition the participant anticipates after having expressed a choice. The comparison of the “self” and “other” conditions during the anticipation phase is complicated by this asymmetry, and must be interpreted with caution. We therefore decided to focus primarily on brain responses during the outcome phase, which has a better design symmetry.

Previous studies in adolescents suggested that there might be developmental differences in cingulate activity following rejection. Several studies have reported that the subgenual part of ACC (sgACC) is preferentially activated during social rejection in adolescence^39–42^, while other studies have found also found activation of the dorsal ACC under these conditions^35,43^. Our finding in dACC is consistent with the latter observations. The studies that found sgACC activity used the Cyberball task, mostly in early adolescent samples. A possible reasons for the discrepancy across findings is therefore that is due to design differences. Unlike the Cyberball game, our online game includes viewing pictures of oneself and explicit social judgments from and toward other players. These and other factors specific to our paradigm may account for differences in the location of cingulate activation observed. In addition, the period of adolescence studied could also contribute to this difference. Most of the studies that found sgACC activation included early adolescent samples. Additional investigations will be required to clarify whether the somewhat inconsistent cingulate response findings relate to developmental effects, design features or both.

The default-mode network (DMN), which includes the dorsomedial prefrontal and the posterior cingulate cortices, is thought to support self-reflection and encoding of stimuli from an egocentric perspective^44^. It could therefore have been expected that self-relevant stimuli, such as the information on social evaluation received in our task, would be associated with activation of these structures. In fact, we did not find greater DMN activity for the self-versus-other distinction. This could be because our online game is relatively fast-paced and interactive, which makes it difficult for subjects to disengage from the game. We propose that the lack of DMN-related findings in the present study is the result of the game prompting a “reactive” versus elaborative mode of self-relevant information processing.

Since the seminal report that demonstrated an overlap between neural substrates of social and physical pain^17,45^, this overlap has been the subject of numerous studies. Some of these have used both social rejection and acute painful stimulation in the same individual to directly assess the overlap in neural activation. Using this approach, activation common to physical and psychological pain was found in the AI and dACC, but also in discriminative brain areas, such as posterior insula (PI) and secondary somatosensory cortex (SII)^46^. Another study used a romantic rejection paradigm in which the participants re-lived an unwanted rejection by a romantic partner, and found similar results^38^.

However, a more fine-grained multi-voxel pattern analysis has suggested distinct affective responses to physically and psychologically painful experiences^26^. Also, challenging the formulation that dACC is simply a “pain region”, another study showed that dACC activity was related to expectancy violation more than to the quality of social feedback^27^. Additional questions are raised by meta-analyses^14–16^. One of these found that the AI was indeed activated bilaterally in social pain paradigms, but did not provide support for a reliable dACC activation in this context^14^. Another recent meta-analysis that included data from 40 whole-brain investigations of social pain failed to identify reliable responses in either AI or dACC^16^. Finally, when detected, rejection-related neural responses tend to be distributed broadly along the medial cortical wall, ranging from subgenual to posterior portions of cingulate cortex, and extending into pre-motor regions^15,17^. Collectively, these data prompt a need to qualify the view that physical and social pain are subserved by common neural substrates in AI and dACC.

In prior studies, social exclusion has been the most if not the only salient condition included. For instance, in the Cyberball task, receiving the ball is the expected outcome, while being excluded is the salient event that violates expectations, making it difficult to ascribe reported neural-functional effects to exclusion versus expectancy violation. Likewise, re-experiencing a recent breakup has a high salience, and is difficult to contrast against an equally salient positive condition. Similar to Dalgleish *et al*.^35^, we achieved balanced salience across acceptance and rejection conditions by ensuring that each trial was independent of the previous as well as of the following trials. In addition, our study included a previously uninvestigated dimension which we believe allowed us to arrive at novel insights with respect to the neural correlates of social interaction. The inclusion of the “self” and “other” distinction showed that AI and dACC activated when the participants received the feedback, irrespective of its quality. This property of dACC and AI only became apparent through the comparison of “self” versus “other” epochs made possible by our design. These findings sheds new light on the involvement of the AI and dACC in social cognition, and strongly argues against a selective role of these two structures as social-pain-specific areas.

Although activated during social or physical pain, the AI and the dACC have been implicated in a variety of other functions, including the processing of homeostatically relevant information and resulting actions. It has been suggested that these structures are part of a “salience network” that monitors the relevance of internal and external inputs, switches brain activity from introspective to externally oriented, and influences action selection^31–33,47,48^. Adequately attributing salience—i.e. paying attention to and taking into account positive as well negative social feedback—is essential for adaptive social behavior. Consistent with this point, gray matter loss in the AI has been found in psychiatric conditions characterized by maladaptive social behaviors, such as alcohol addiction and frontal dementia^49,50^.

We hypothesized that a key property of social evaluation is whether it applies to oneself or others, and that the former is what makes social information salient. In agreement with our hypothesis, rather than reflecting responses to negative social evaluation, the activation of dACC and AI was related to the monitoring of self-relevant information, irrespective of the valence of the social feedback. This observation is in agreement with, and expands on recent findings^35^, by showing that activation of dACC and AI are not specific to social rejection or acceptance. Our findings do not address the question whether dACC and AI are selectively involved in processing social information, since we did not compare responses to social vs non-social stimuli. However, we do propose that in the context of social interactions, these regions are encoding the salience of the self. It is important to note, however, that we intentionally decided not to manipulate the quality of the feedback across self and other conditions in order to provide the same contextual frame. By keeping the salience level of the feedback equal across conditions we were able to show that self-relational processing coincided with higher salience processing. This might not be the case in designs in which the balance of the quality of the social feedback is not guaranteed. Manipulation of the salience of the social feedback was, however, beyond the scope of our study.

In summary, our data suggest that a pattern of brain responses to social acceptance and rejection critically depends on their context as self-relevant or not. The structures involved in these responses, AI and dACC, can therefore be conceptualized in monitoring the social salience of the self, such that their activation encodes the high relevance of being judged by others. This supports an emerging reinterpretation of AI and dACC as components of a “social pain matrix.”

## Materials and Methods

### Participants

Forty adolescents (20 females) were recruited via advertisement in schools and on Facebook. The age of the participants ranged from 15 to 19 (17.2 ± 1.39; mean ± SD). Participants had no history of mental illness or any history of seeking psychological or psychiatric support, as determined by an e-mail interview prior to the experiment. Informed consent was provided by the participants, and for participants under 18 years of age, also by the parents. The study was approved by the Linköping Regional Ethics Board (Dnr 2015-273-31), and was carried out according to the declaration of Helsinki.

### The online game

Participants engaged in a simulated online game in which they decided and indicated whether they liked or disliked pictures of other adolescents. Similarly, other putative players also judged the participants’ pictures. The pictures consisted of neutral frontal face photos. Each trial of this rapid event-related design consisted of three epochs: the question phase, the anticipation phase and the outcome phase. During the question phase of “other” trials, a picture of an adolescent was shown on the screen for 3 seconds with the question “Do you like him?” or “Do you like her?”. The participant was instructed to choose whether he/she liked this person by pressing a button with their index or middle finger for “yes” and “no” answers respectively. After providing a choice, a thumbs-up or a thumbs-down feedback image appeared to the left of the picture. The thumbs-up picture indicated that the participant chose “yes”, whereas the thumbs-down picture indicated that the participant chose “no” (see Fig. 1a). Afterwards the word “calculating” was displayed over the picture for 2000–4000 milliseconds. This interval represented the anticipation phase of the trial and signaled that the average rating of the participant and of a few other putative players was being calculated. In order to optimize the estimation of the impulse response functions (IRF), the anticipation phase was jittered in 2000, 3000, or 4000 milliseconds. By using integer values we could guarantee that the end of our stimuli coincided with either a full or a half TR. Finally, during the outcome phase of the trial, the collective outcome was superimposed over the picture and shown for 3 seconds. In order to minimize unmodeled neural effects induced by potential conflict monitoring, the final outcome matched the subject’s choice. The pictures of the putative players were different across runs and were taken from the website www.shutterstock.com. In order to eliminate expectation effects the pictures were not shown to the subjects beforehand and depicted strangers.

During “self” trials the participant’s picture was shown and rated by a few participants and these participants changed from trial to trial. The subject did not know who was doing the rating. The participant saw his/her own picture with the question “Do they like you?” and waited until the positive or negative feedback cue appeared over their picture. The participant took a picture of himself/herself for use in the online game at the end of the training session described below. Each of two runs of the online game consisted of 16 “self” and 16 “other” trials, for a total of 32 total trials per condition. Within the “self” condition the number of positive- and negative-feedback trials was balanced (8 likes and 8 dislikes per run, 16 per condition in total) and randomly presented. The jittered inter-trial interval varied between 2000, 3000 or 4000 milliseconds. The sequence was counterbalanced so that “self” and “other” conditions were not presented more than twice consecutively. In addition, we counterbalanced the order in which females and males players were displayed. See Fig. 1 for a schematic of the study structure.

After the scan the participants were asked to answer the following on a visual analogue scale (VAS): (1) How often were you disliked? (0% “never”−100% “always”); (2) How much did you like to see your own face? (0 “not at all”−10 “very much”); (3) How bad did it feel to be disliked? (0 “not at all” – 10 “very much”); and (4) How good did it feel to be liked? (0 “not at all” – 10 “very much”).

### Questionnaires

To ensure group homogeneity and that the participants had no severe psychiatric symptoms or extreme personality traits, participants were asked to complete two questionnaires: the Strengths and Difficulties Questionnaire [SDQ^51^;] and the Health-Relevant Personality Inventory [HP5i^52^;]. The SDQ is an established psychiatric screening form and has 25-items divided in five subscales: emotional symptoms, conduct problems, hyperactivity/inattention, peer relations and pro-social behaviours. The HP5i is a 20-items questionnaire based on the Big Five model, which assesses the following personality traits: Openness, Conscientiousness, Extraversion, Agreeableness and Neuroticism. The items consist of short phrases such as “choosing rapidly with little thought” and “acting on the spur of the moment” for the factor of conscientiousness. Questionnaire results are described in the Supplementary information.

### Training session

Before the magnetic resonance imaging (MRI) session, participants underwent a training session in an MR simulator system (PST MR Simulator System, BlindSight GmbH, Schlitz, Germany). During the training session, participants habituated to the MRI environment and were trained to lie still via feedback from a motion tracking system positioned around their head (MoTrak Head Motion Tracking System, Psychology Software Tools, Sharpsburg, PA, USA). In addition, the participants received instructions and did a trial run of the task. In the trial version of the task, pictures depicting head silhouettes were used instead of pictures of real people. Finally, the participants took a picture of themselves using a smartphone. They were instructed to keep a neutral facial expression and were left alone during that time. In total, the training session took approximately 45 minutes.

### MRI Data Acquisition

Imaging was performed using a Philips Ingenia 3 Tesla MR scanner (Philips Healthcare, Best, The Netherlands) equipped with a 32-channel Philips dS Head head-coil. Six dummy volumes were acquired before each scan to allow the spin system to reach steady-state longitudinal magnetization and reduce possible effects of partial saturation. Blood oxygen-level-dependent (BOLD) data were acquired with an echo-planar imaging (EPI) sequence: TR = 2000 ms; TE = 30 ms; flip angle = 77°; field-of-view = 220 × 220; in-plane resolution = 3.4 × 3.4 mm; slice thickness = 4 mm, no slice gap; number of axial slices (angled with the AC-PC line) = 32; number of volumes = 195. Two functional runs were collected and each run lasted for 6 minutes and 45 seconds. A high-resolution 3D T1-weighted Turbo Field Echo scan was acquired before the EPI data acquisitions: TR = 7.0 ms; TE = 3.2 ms; flip angle = 8°; field-of-view = 256 × 240 × 170 mm; voxel resolution = 1 × 1 × 1 mm; no slice gap; plane: sagittal; number of sagittal slices = 170. Heart rate and respiration were monitored using a peripheral pulse unit (PPU) and a pneumatic respiration transducer belt respectively (SpO2 MRI sensor, Invivo, Orlando, FL, USA).

### fMRI Data Preprocessing

Preprocessing was performed with the Analysis of Functional Neuro Images (AFNI) software v16.2.12^53^. BOLD images were de-spiked and slice-time corrected. For motion correction and co-registration purposes each EPI volume was registered to the volume with the minimum outlier fraction (using the AFNI outlier definition). Functional images were then warped to Talairach template space using a combination of affine and non-linear transformations^54^. Nuisance effects due to head motion (estimated from the motion correction procedure) were accounted for by adding the motion parameters (and their derivatives) as regressors of no interest in the main regression. A motion censoring threshold of 0.3 mm per TR was implemented in combination with an outlier fraction threshold of 0.1. Volumes violating either of these thresholds were subsequently ignored in the time-series regression. Nuisance effects from physiological processes (heart beat and respiration) were also addressed in the regression using both AFNIs RICOR function and regressors generated by the RVHRCOR method^55,56^.

### fMRI Data Analysis

A general linear model (GLM) analysis was performed to capture differences across conditions. A unique input stimulus function was defined for each task period. Input stimulus functions were convolved with the AFNI gamma hemodynamic response function to yield regressors for the GLM. Whole-brain, voxel-wise GLM statistical analysis was carried out on the BOLD time-series data using 3dDeconvolve. We conducted GLM-based analysis of the anticipation phase and the outcome phase for “self” and “other” conditions (Fig. 1). For the outcome phase, we included additional regressors modeling positive (like) or negative (dislike) feedback conditions. We included an additional regressor of no interest modelling effects of motor response (every instance the subject pressed any of the buttons) on BOLD time-series data. To determine significance of effects at the group level, we conducted a within-subject *t*-test on response estimates for the “anticipation” interval for “self” versus “other” conditions. Moreover, we conducted a 2 × 2 factorial analysis of variance with factors “Perspective” (2 levels: self and other) and “Outcome” (levels: rejection, acceptance) during the outcome interval. We used the AFNI program 3dClustSim to determine cluster-size thresholds necessary for identifying effects significant at *alpha* = 0.05 family-wise-error corrected. Average spatial smoothness estimates, across all participants, used by 3dClustSim were obtained using the 3dFWHMx function with the ACF flag, as per current recommendations from the maintainers of AFNI^57–59^.

### Data availability

The datasets generated during and/or analysed during the current study are available from the corresponding author on reasonable request.

## Electronic supplementary material

Supplementary information -Questionnaires results.

## References

[1] Steinberg L (2005). Cognitive and affective development in adolescence. Trends Cogn Sci.

[2] Higley JD (1996). Excessive mortality in young free-ranging male nonhuman primates with low cerebrospinal fluid 5-hydroxyindoleacetic acid concentrations. Arch. Gen. Psychiatry.

[3] Gruenenfelder-Steiger AE, Harris MA, Fend HA (2016). Subjective and objective peer approval evaluations and self-esteem development: A test of reciprocal, prospective, and long-term effects. Dev Psychobiol.

[4] Luna B, Padmanabhan A Fau - O’Hearn K, O’Hearn K (2010). What has fMRI told us about the development of cognitive control through adolescence?. Brain Cogn.

[5] Burnett S, Sebastian C, Cohen Kadosh K, Blakemore SJ (2011). The social brain in adolescence: evidence from functional magnetic resonance imaging and behavioural studies. Neurosci Biobehav Rev.

[6] Williams, K. D. *Ostracism: The power of silence* (Guilford Press, 2002).

[7] Leary MR, Tambor ES, Terdal SK, Downs DL (1995). Self-esteem as an interpersonal monitor: The sociometer hypothesis. Journal of personality and social psychology.

[8] Baumeister RF, Twenge JM, Nuss CK (2002). Effects of social exclusion on cognitive processes: anticipated aloneness reduces intelligent thought. Journal of personality and social psychology.

[9] Heilig M, Epstein DH, Nader MA, Shaham Y (2016). Time to connect: bringing social context into addiction neuroscience. Nature reviews. Neuroscience.

[10] Williams KD, Cheung CK, Choi W (2000). Cyberostracism: effects of being ignored over the Internet. Journal of personality and social psychology.

[11] Williams KD, Jarvis B (2006). Cyberball: A program for use in research on interpersonal ostracism and acceptance. Behavior research methods.

[12] Gonsalkorale K, Williams KD (2007). The KKK won’t let me play: Ostracism even by a despised outgroup hurts. European Journal of Social Psychology.

[13] Zadro L, Williams KD, Richardson R (2004). How low can you go? Ostracism by a computer is sufficient to lower self-reported levels of belonging, control, self-esteem, and meaningful existence. Journal of Experimental Social Psychology.

[14] Cacioppo S (2013). A quantitative meta-analysis of functional imaging studies of social rejection. Sci Rep.

[15] Rotge JY (2015). A meta-analysis of the anterior cingulate contribution to social pain. Soc Cogn Affect Neurosci.

[16] Vijayakumar, N., Cheng, T. W. & Pfeifer, J. H. Neural correlates of social exclusion across ages: A coordinate-based meta-analysis of functional MRI studies. *Neuroimage* (2017). 10.1016/j.neuroimage.2017.02.050 PMC5457721 28235565

[17] Eisenberger NI, Lieberman MD, Williams KD (2003). Does rejection hurt? An FMRI study of social exclusion. Science.

[18] Peyron R, Laurent B, Garcia-Larrea L (2000). Functional imaging of brain responses to pain. A review and meta-analysis (2000). Neurophysiol Clin.

[19] Macdonald G, Leary MR (2005). Why does social exclusion hurt? The relationship between social and physical pain. Psychological bulletin.

[20] Casey KL (2000). Selective opiate modulation of nociceptive processing in the human brain. J Neurophysiol.

[21] Price D, V der Gruen A, Miller J, Rafii A, Price C (1985). A psychophysical analysis of morphine analgesia. Pain.

[22] Goodin BR, Ness TJ, Robbins MT (2015). Oxytocin - a multifunctional analgesic for chronic deep tissue pain. Current pharmaceutical design.

[23] Panksepp J, Herman B, Conner R, Bishop P, Scott J (1978). The biology of social attachments: Opiates alleviate separation distress. Biological psychiatry.

[24] Swain JE, Mayes LC, Leckman JF (2005). Endogenous and exogenous opiates modulate the development of parent-infant attachment. Behavioral and Brain Sciences.

[25] Panksepp J, Nelson E, Bekkedal M (1997). Brain Systems for the Mediation of Social Separation‐Distress and Social‐Reward Evolutionary Antecedents and Neuropeptide Intermediariesa. Annals of the New York Academy of Sciences.

[26] Woo CW (2014). Separate neural representations for physical pain and social rejection. Nature communications.

[27] Somerville LH, Heatherton TF, Kelley WM (2006). Anterior cingulate cortex responds differentially to expectancy violation and social rejection. Nat Neurosci.

[28] Mouraux A, Diukova A, Lee MC, Wise RG, Iannetti GD (2011). A multisensory investigation of the functional significance of the “pain matrix”. Neuroimage.

[29] Iannetti GD, Salomons TV, Moayedi M, Mouraux A, Davis KD (2013). Beyond metaphor: contrasting mechanisms of social and physical pain. Trends Cogn Sci.

[30] Shackman AJ (2011). The integration of negative affect, pain and cognitive control in the cingulate cortex. Nature reviews. Neuroscience.

[31] Uddin LQ (2015). Salience processing and insular cortical function and dysfunction. Nat Rev Neurosci.

[32] Menon V, Uddin LQ (2010). Saliency, switching, attention and control: a network model of insula function. Brain structure & function.

[33] Goulden N (2014). The salience network is responsible for switching between the default mode network and the central executive network: replication from DCM. Neuroimage.

[34] Sridharan D, Levitin DJ, Menon V (2008). A critical role for the right fronto-insular cortex in switching between central-executive and default-mode networks. Proc. Natl. Acad. Sci. USA.

[35] Dalgleish T (2017). Social pain and social gain in the adolescent brain: A common neural circuitry underlying both positive and negative social evaluation. Sci Rep.

[36] Bale TL, Epperson CN (2017). Sex as a Biological Variable: Who, What, When, Why, and How. Neuropsychopharmacology.

[37] Nordicom-Sveriges. Mediebarometer. (Gothenburg, 2017).

[38] Kross E, Berman MG, Mischel W, Smith EE, Wager TD (2011). Social rejection shares somatosensory representations with physical pain. Proc Natl Acad Sci USA.

[39] Masten CL (2009). Neural correlates of social exclusion during adolescence: understanding the distress of peer rejection. Soc Cogn Affect Neurosci.

[40] Bolling DZ, Pelphrey KA, Vander Wyk BC (2016). Unlike adults, children and adolescents show predominantly increased neural activation to social exclusion by members of the opposite gender. Soc Neurosci.

[41] Masten CL (2011). Subgenual anterior cingulate responses to peer rejection: a marker of adolescents’ risk for depression. Development and psychopathology.

[42] van Harmelen AL (2014). Childhood emotional maltreatment severity is associated with dorsal medial prefrontal cortex responsivity to social exclusion in young adults. PLoS One.

[43] Masten CL, Telzer EH, Fuligni AJ, Lieberman MD, Eisenberger NI (2012). Time spent with friends in adolescence relates to less neural sensitivity to later peer rejection. Soc Cogn Affect Neurosci.

[44] Spreng RN, Mar RA, Kim AS (2009). The common neural basis of autobiographical memory, prospection, navigation, theory of mind, and the default mode: a quantitative meta-analysis. J Cogn Neurosci.

[45] Eisenberger NI (2012). The pain of social disconnection: examining the shared neural underpinnings of physical and social pain. Nat Rev Neurosci.

[46] Novembre G, Zanon M, Silani G (2015). Empathy for social exclusion involves the sensory-discriminative component of pain: a within-subject fMRI study. Soc Cogn Affect Neurosci.

[47] Seeley WW (2007). Dissociable intrinsic connectivity networks for salience processing and executive control. J Neurosci.

[48] Craig AD (2009). How do you feel–now? The anterior insula and human awareness. Nature reviews. Neuroscience.

[49] Seeley WW (2010). Anterior insula degeneration in frontotemporal dementia. Brain structure & function.

[50] Senatorov VV (2015). Reduced anterior insula, enlarged amygdala in alcoholism and associated depleted von Economo neurons. Brain.

[51] Lundh LG, Wångby-Lundh M, Bjärehed J (2008). Self-reported emotional and behavioral problems in Swedish 14 to 15-year-old adolescents: a study with the self-report version of the Strengths and Difficulties Questionnaire. Scandinavian journal of psychology.

[52] Gustavsson JP, Jönsson EG, Linder J, Weinryb RM (2003). The HP5 inventory: definition and assessment of five health-relevant personality traits from a five-factor model perspective. Personality and Individual Differences.

[53] Cox RW (1996). AFNI: software for analysis and visualization of functional magnetic resonance neuroimages. Computers and biomedical research, an international journal.

[54] Talairach, J. & Tournoux, P. *Co-Planar Stereotaxic Atlas of the Human Brain* (Thieme Medical Publishers, New York, 1988).

[55] Chang C (2013). Association between heart rate variability and fluctuations in resting-state functional connectivity. Neuroimage.

[56] Glover GH, Li TQ, Ress D (2000). Image-based method for retrospective correction of physiological motion effects in fMRI: RETROICOR. Magn Reson Med.

[57] Eklund A, Nichols TE, Knutsson H (2016). Cluster failure: Why fMRI inferences for spatial extent have inflated false-positive rates. Proc Natl Acad Sci USA.

[58] Cox RW, Chen G, Glen DR, Reynolds RC, Taylor PA (2017). FMRI Clustering in AFNI: False-Positive Rates Redux. Brain connectivity.

[59] Cox RW, Chen G, Glen DR, Reynolds RC, Taylor PA (2017). fMRI clustering and false-positive rates. Proc Natl Acad Sci USA.

