# Supplementary material for: The salience of self, not social pain, is encoded by dorsal anterior cingulate and insula
Source: Sci Rep. 2018 Apr 18;8:6165. doi: 10.1038/s41598-018-24658-8 (PMC5906579; doi:10.1038/s41598-018-24658-8)
Supplement: Supplementary file 1 — Supplementary information -Questionnaires results. [file 41598_2018_24658_MOESM1_ESM.docx]

**Title: The salience of self, not social pain, is encoded by dorsal anterior cingulate and insula.**

Irene Perini^*^, Per A. Gustafsson, J. Paul Hamilton, Robin Kämpe, Maria Zetterqvist, and Markus Heilig

Center for Social and Affective Neuroscience, Department of Clinical and Experimental Medicine, Linköping University, 581 83 Linköping, Sweden

^*^Corresponding author. [irene.perini@liu.se](mailto:irene.perini@liu.se)

**Supplementary information**

**Questionnaires results.** Measures of personality traits were largely within the range expected for Swedish adolescents, although some differences were noted. Specifically, participants reported being significantly more prosocial (*p* = 0.024) than the normative Swedish group, with females also having significantly lower oppositional traits (SDQ questionnaire: *p* = 0.005). Compared to males, females rated themselves as having more emotional symptoms (*p* = 0.041) and less hyperactivity (*p* = 0.008). Participants scored significantly higher on Extraversion compared to Swedish norms (*p* = 0.007 for females, *p* =0.003 for males). In addition females rated themselves as significantly more agreeable (*p* = 0.045), more conscientious (*p* = 0.027) and with lower Neuroticism (*p* = 0.022) compared to the normal sample. Males were more prone to describe themselves as less agreeable (*P* = 0.033) and Conscientious (*p* = 0.025) compared to females.
